# Generation of Gαi knock-out HEK293 cells illuminates Gαi-coupling diversity of GPCRs

**DOI:** 10.1038/s42003-023-04465-2

**Published:** 2023-01-28

**Authors:** Yuki Ono, Kouki Kawakami, Gaku Nakamura, Satoru Ishida, Junken Aoki, Asuka Inoue

**Affiliations:** 1 Molecular and Cellular Biochemistry, Graduate School of Pharmaceutical Sciences, Tohoku University, Sendai, Miyagi 980-8578 Japan; 2 Department of Health Chemistry, Graduate School of Pharmaceutical Sciences, The University of Tokyo, Bunkyo-ku, Tokyo 113-0033 Japan

**Keywords:** Pharmacology, Sensors and probes

## Abstract

G-protein-coupled receptors (GPCRs) are pivotal cell membrane proteins that sense extracellular molecules and activate cellular responses. The G-protein α subunit i (Gαi) family represents the most common GPCR-coupling partner and consists of eight subunits with distinct signaling properties. However, analyzing the coupling pattern has been challenging owing to endogenous expression of the Gαi subunits in virtually all cell lines. Here, we generate a HEK293 cell line lacking all Gαi subunits, which enables the measurement of GPCR-Gαi coupling upon transient re-expression of a specific Gαi subunit. We profile Gαi-coupling selectivity across 11 GPCRs by measuring ligand-induced inhibitory activity for cAMP accumulation. The coupling profiles are then classified into three clusters, representing those preferentially coupled to Gαz, those to Gαo, and those with unapparent selectivity. These results indicate that individual Gαi-coupled GPCRs fine-tune Gαi signaling by exerting coupling preference at the Gαi-subunit level.

## Abstract (web summary)

A HEK293 cell line lacking all Gαi subunits is generated which enables the measurement of coupling between a GPCR and individual Gαi subunits upon transient re-expression of a specific Gαi subunit.

## Introduction

G-protein-coupled receptors (GPCRs), the key transducers that link extracellular stimuli to downstream intracellular signals, are the most common targets for drug development^1^. Upon GPCR ligand binding, the heteromeric guanine nucleotide-binding proteins (G proteins), consisting of α, β, and γ subunits, induce activation of their effector proteins. Among the three subunits, the α subunit mainly determines the key properties of individual heterotrimeric G proteins. The human genome encodes 16 α subunits, which are grouped into four subfamilies based on their sequence homology and functional similarity, namely, Gαs, Gαi, Gαq, and Gα12^2^. The Gαi subfamily is the most common coupling partner in the family A GPCRs^3,4^. Furthermore, approximately 45% of GPCRs targeted by the United States Food and Drug Administration (FDA)-approved drugs couple to the Gαi subfamily^5^, highlighting the significance of Gαi-coupled GPCRs as drug targets.

Previous studies have shown that the Gαi subunits have both overlapping and distinct functions. The Gαi subfamily consists of eight α subunits: Gαi1, Gαi2, Gαi3, Gαt1, Gαt2, Gαt3, Gαo, and Gαz. Activation of the Gi heterotrimer induces various cellular signaling processes such as the inhibition of adenylyl cyclase and calcium channels, and the activation of potassium channels^6^. Despite the high sequence homology within the Gαi subfamily, several subunit-specific functions have been reported. For example, transducin (Gαt1 and Gαt2) and gustducin (Gαt3) are involved in visual and taste perception, respectively, both by activating cGMP phosphodiesterase^7,8^. Additionally, using Gαi subunit knockdown experiments in GH4C1 cells, Gαo and Gαi2 were found to mediate inhibition of calcium entry and cAMP accumulation, respectively^9^. The Gαi subunits also behave biochemically different from one another. It should be noted, however, that these biochemical properties could be modified in living cells as observed in Gq, which shows slower nucleotide dissociation in purified condition than in lipid membrane condition. For example, the rate of spontaneous GDP dissociation from Gαo is an order of magnitude faster than that of Gαi1-3^10^, and the intrinsic GTPase activity of Gαz is 200-fold slower than that of the other Gαi subunits^11^. These differences between the members of the Gαi family allow for diverse Gαi-coupled GPCR signal transduction.

Although signaling properties of the Gαi subunits have been well characterized, Gαi-subunit-coupling selectivity of GPCRs has only been investigated for a limited number of receptors due to experimental difficulties. Individual GPCR-Gαi coupling cannot be assessed simply by expressing a Gαi subunit of interest because virtually all mammalian cells endogenously express multiple Gαi subunits^12^. To eliminate endogenous GPCR-Gαi coupling, previous studies have used pertussis toxin (PTX), which ADP-ribosylates the cysteine residue in the C-terminal tail of the Gαi subunits^13^. By expressing a PTX-resistant Gαi mutant with a substitution at the cysteine residue, PTX treatment enables the measurement of selective coupling of the mutant Gαi subunit^14–16^. However, altering the cysteine residue can affect the G-protein coupling abilities of the α subunits^17,18^. Additionally, PTX does not inhibit Gαz since it has isoleucine at the PTX-targeting site^19^. Recently, fluorescence or bioluminescence resonance energy transfer-based techniques have been used to evaluate coupling of individual engineered Gα subunits^20,21^. However, these methods require the insertion of luciferase or a fluorescent protein into the α subunit, and such modification affects its biochemical properties and resulting coupling efficiency^22^.

In this study, we circumvented the experimental difficulties in measuring individual intact Gαi subunits by employing genome-editing approaches to establish a Gαi-null background in human embryonic kidney (HEK) 293A cells. By transiently expressing a pair of a GPCR and a Gαi subunit of interest into the Gαi-deficient HEK293A cells, we successfully characterized Gαi-coupling profiles in 11 GPCRs and found physiologically reasonable selectivity of the Gαi subunits for these GPCRs.

## Results

### Generation of a HEK293A cell line lacking Gαi subunits

**Fig. 1:**
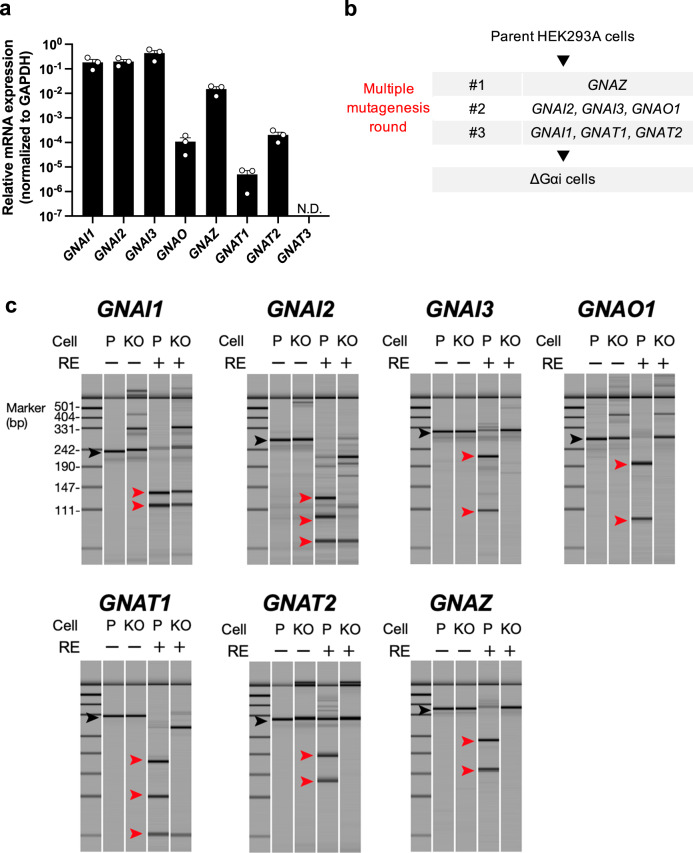
Generation of the ∆Gαi HEK293A cell line. **a** Quantitative real-time PCR analysis of the Gαi subunits in the parent HEK293A cells. Bars and error bars represent the mean and standard error of the mean (SEM), respectively, of three independent experiments. Each experiment was performed in duplicate. Abbreviation: N.D., not detected. **b** Strategy for genetic inactivation of the Gαi family members. ∆Gαi cells were obtained using three-round CRISPR–Cas9 mutagenesis. **c** Identification of the Gαi-mutant clone genotype using the restriction-enzyme digested-fragment method. The sgRNA targets were PCR-amplified and treated with their corresponding restriction enzymes (RE). Digested samples were subjected to capillary electrograph analysis, and pseudo-gel images of the electropherogram were visualized with a DNA marker of the pUC19/Hpa II digest. Black and red arrowheads indicate undigested and RE-digested PCR fragments of the targeted site, respectively. Note that there are two RE sites in PCR fragments of the *GNAI2* and *GNAT1* genes, and one of the mutated alleles in the *GNAI1* gene could be digested by RE. Abbreviations: P parental, KO knock-out.

In order to establish a Gαi-null background for functional analysis of Gαi subunit-coupling patterns, we generated a HEK293A cell line devoid of all Gαi subunits. First, we measured expression of the Gαi subunits in HEK293A cells by quantitative RT-PCR analysis. While seven Gαi subunit-encoding genes (*GNAI1*, *GNAI2*, *GNAI3*, *GNAO1*, *GNAT1*, *GNAT2*, and *GNAZ*) were expressed, *GNAT3* expression was undetectable (Fig. 1a). To validate the PCR primer pair for *GNAT3*, we measured and confirmed *GNAT3* expression in a sample of small intestine tissue, in which *GNAT3* is known to be expressed^23^ (Supplementary Fig. 1). We next targeted the seven Gαi subunit-encoding genes present in HEK293A cells using three-round CRISPR–Cas9 mutagenesis (Fig. 1b). We designed single-guide RNAs (sgRNAs) to induce double-stranded DNA cleavage within the N-terminal half of each α-subunit. Upon successful introduction of frameshift or nonsense mutations by these sgRNAs, mutated genes are expected to produce truncated Gαi subunits with the elimination of the c-terminal α5 helix, which is responsible for receptor coupling^24^. After transfection of the sgRNA constructs, fluorescence-activated cell sorting isolation of GFP-positive cells, and expansion of the clonal cell colonies, the clones were screened for mutations in the target genes using the restriction-enzyme method, obtaining a knock-out clone candidate (ΔGαi cells) (Fig. 1c). Sanger sequencing of the target genomic regions confirmed that the ΔGαi cells carry frameshift mutations or large insertions, including stop codons, in all targeted alleles (Supplementary Fig. 2). *GNAT3* expression remained undetected in the ΔGαi cells (Supplementary Fig. 1). These results indicate that the seven Gαi subunit-encoding genes were successfully mutated in the HEK293A cells to prevent their endogenous expression.

**Fig. 2:**
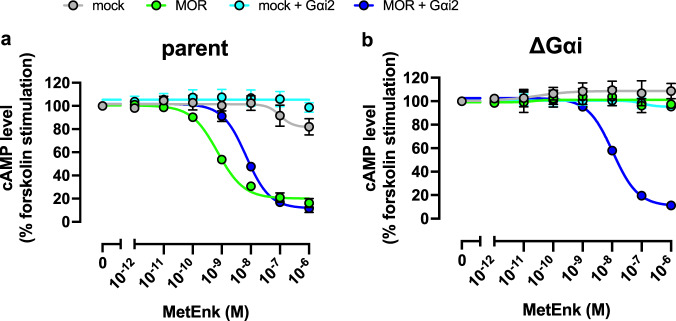
Functional validation to verify successfully knocking out the Gαi subunits. **a**, **b** Concentration–response curves of the GloSensor cAMP assay in parent (**a**) and ∆Gαi cells (**b**). Parent and ∆Gαi cells transiently expressing Glo-22F cAMP reporter with MOR and Gαi2 were stimulated with 10 µM forskolin along with indicated concentrations of MetEnk. For each experiment, forskolin-induced cAMP accumulation was set as 100%. In both figures, symbols and error bars represent the mean and SEM, respectively, of three independent experiments. Each experiment was performed in triplicate. Note that for many data points, error bars are smaller than the size of the symbols, and thus are not visible.

Next, we functionally examined the lack of Gαi subunits in ΔGαi cells using a GloSensor cAMP assay. We expressed µ-opioid receptor (MOR), a prototypical Gαi-coupled receptor, along with the GloSensor cAMP reporter and measured cAMP levels upon stimulation with the ligand, methionine-enkephalin (MetEnk), together with forskolin. In the parent cells, forskolin-stimulated cAMP accumulation was almost completely inhibited in a MetEnk concentration-dependent manner (Fig. 2a). In contrast, the ∆Gαi cells were completely unresponsive to MetEnk, while exogenous Gαi2 expression restored their response to the ligand (Fig. 2b). These results show that the ΔGαi cells are devoid of functional Gαi subunits, making them suitable for investigating Gαi subunit signaling.

To further characterize the ∆Gαi cells, we compared cell proliferation of the parent and ∆Gαi cells. We assessed cell proliferation by collecting the cells 48 h after seeding and counting the number of cells. Cell proliferation of the ∆Gαi cells in 48 h was reduced by 20% compared to that of parent cells (Supplementary Fig. 3). Similar to the ∆Gαi cells, PTX-treated parent cells also showed a 20% reduction in cell proliferation compared to that of non-treated parent cells. This result suggests that the suppression of cell proliferation in the ∆Gαi cells is not due to a clonal effect but to the on-target deficiency of Gαi proteins. In addition, the reduced proliferation in ∆Gαi cells and the PTX-treated parent cells were both recovered by culturing the cells in type I collagen-coated dishes (Supplementary Fig. 3), suggesting that the suppression of cell proliferation is due to the weakened adhesion of the ∆Gαi cells.

**Fig. 3:**
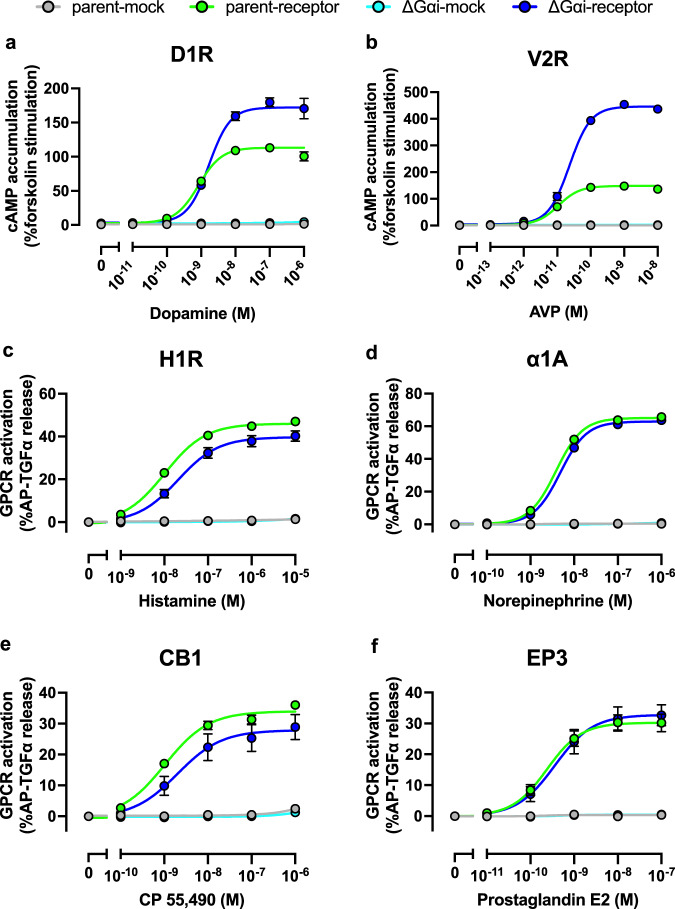
Assessing Gs, Gq, and G12 signaling in ∆Gαi cells. **a**, **b** Concentration-response curves of Gs-mediated cAMP accumulation. Parent and ∆Gαi cells transiently expressing Glo-22F together with either D1R (**a**) or V2R (**b**) were stimulated with the indicated concentrations of dopamine or arginine vasopressin (AVP), respectively. **c**–**f** Concentration–response curve of the TGFα shedding responses induced by Gq- (H1R (**c**) and α1A (**d**)) and G12-coupled receptor (CB1 (**e**) and EP3 (**f**)) activation in parent and the ∆Gαi cells. A test GPCR was expressed in the parent and the ∆Gαi cells along with the AP-TGFα reporter, and the resulting ligand-induced response was assessed. In all figures, symbols and error bars represent the mean and SEM, respectively, of three independent experiments. Each experiment was performed in triplicate. Note that for many data points, error bars are smaller than the size of the symbols, and thus are not visible.

To examine whether the other G-protein signaling would be affected in the ΔGαi cells, Gs, Gq, and G12 signaling were individually evaluated. We expressed Gs-coupled vasopressin V2 receptor (V2R) or dopamine D1 receptor (D1R) along with the GloSensor cAMP reporter in both the ΔGαi cells and parent cells. The cells were then stimulated with their agonists or forskolin, and measured for their cAMP accumulation. Gs signaling in the ΔGαi cells was enhanced compared to that in the parent cells (Fig. 3a, b). We next measured Gq and G12 signaling via transforming growth factor (TGF) α shedding assay^25^ using Gq- (H1R and α1A) or G12- (EP3 and CB1) coupled GPCRs. By using G-protein-deficient cells, we have previously validated that TGFα shedding responses induced by the tested Gq-GPCR (H1R and α1A) and the tested G12-GPCR (CB1 and EP3) were solely dependent on Gq/11 and G12/13, respectively^3^. Each GPCR was expressed together with an alkaline phosphatase-tagged TGFα (AP-TGFα) reporter in the ∆Gαi cells and the parent cells, and subsequently measured for their ligand-induced AP-TGFα ectodomain shedding response. Gq and G12 signaling in the ΔGαi cells were comparable to that in parent cells (Fig. 3c–f). These data demonstrate that the elimination of the Gαi subunits does not affect Gq and G12 signaling but does potentiate Gs signaling, possibly due to the loss of competition in adenylyl cyclases between Gs and Gi.

We additionally examined whether Gi-coupled GPCRs gain access to other Gα proteins in the absence of Gαi proteins using MOR as an example. As shown in Fig. 2b, MOR activation did not increase cAMP in the ∆Gαi cells, showing that it does not couple to Gs even in the absence of Gαi. We further examined whether MOR couples to Gq and G12 in the Gαi-deficient condition using the TGFα shedding assay. We found that MOR showed no detectable TGFα shedding response in both parent and the ∆Gαi cells (Supplementary Fig. 4), indicating that MOR fails to couple with Gq and G12 even in the absence of Gαi proteins. Of note, we validated activation of MOR in the TGFα shedding assay by using the Gαq/i1 chimeric Gα protein, which binds to Gi-coupled GPCRs and transduces Gq signaling, as a positive control (Supplementary Fig. 4). While these data support that MOR does not couple to non-preferred Gα even in the absence of preferred Gα, we cannot exclude the possibility that other Gi-coupled GPCRs will behave differently. Thus it is recommended to consider this possibility when analyzing G-protein coupling using the Gα-deficient cells.

### Divergent Gαi subunit-coupling preferences across GPCRs

We evaluated GPCR-Gαi-coupling profiles by expressing a pair of GPCR and Gαi subunit of interest in the ∆Gαi cells. We chose five Gαi subunits, namely, Gαi1, Gαi2, Gαi3, Gαo, and Gαz, out of the eight Gαi subunits for the following characterization since physiological effector proteins for the rest of the Gαi subunits (Gαt1, Gαt2, and Gαt3) are known to be cGMP phosphodiesterase.

To determine suitable amounts of the plasmids in the assay, we tested multiple amounts of plasmids encoding GPCR or Gαi subunits and compared concentration-response curves. First, we measured membrane expression levels of MOR, a representative GPCR used in the study, by titrating the MOR-encoding plasmid (100 ng Gαi1 plasmid for all of the MOR conditions). From a plasmid dose of 8–200 ng (per well in a 6-well plate), expression levels increased and its level did not further elevated in the 1000 ng condition (Supplementary Fig. 5a, b). We then measured Gi-mediated cAMP response by the GloSensor assay. The concentration–response curve shifted to the left as the plasmid amount was increased, and plateaued at 200 ng volume (Supplementary Fig. 5c). Therefore, we used 200 ng of the plasmid encoding test GPCRs in the subsequent experiments. In the case of Gα, we fixed the MOR-encoding plasmid as 200 ng and titrated the plasmid amounts of all of the five Gαi subunits from 1 to 1000 ng by 10-fold. A plasmid dose of 1 ng showed negligible cAMP-suppressive effect, while a plasmid dose of 10 ng partially inhibited cAMP production (Supplementary Fig. 5d). Plasmid doses of 100 and 1000 ng almost completely inhibited cAMP production. We note that, from the plasmid volumes of 10 to 1000 ng, the concentration-response curve tended to shift to the right as the plasmid dose increased. Together, subsequent experiments were performed with a Gαi-encoding plasmid dose of 100 ng.

**Fig. 4:**
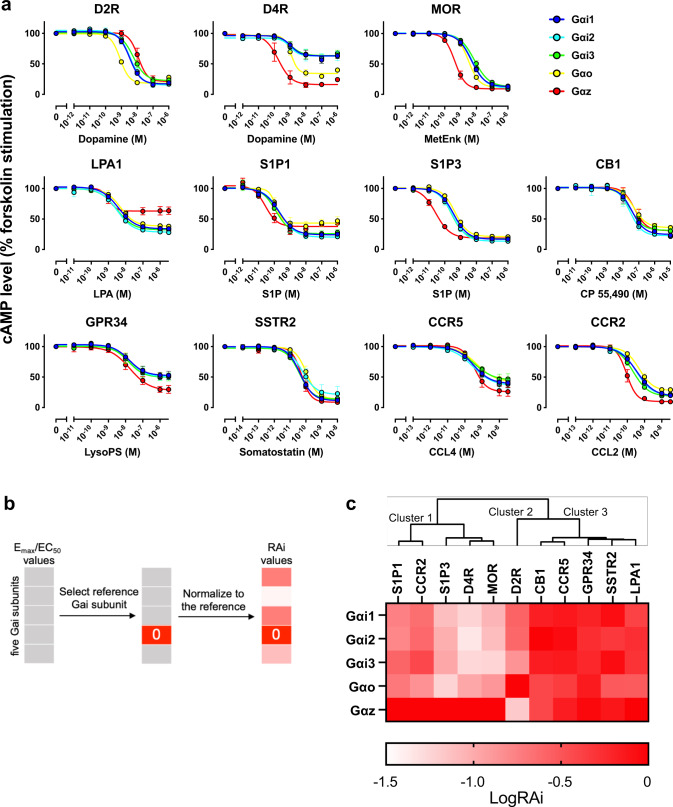
Gαi subunit-coupling profile in ∆Gαi cells as determined by GloSensor cAMP assay. **a** Concentration–response curves of GloSensor cAMP assays in the ∆Gαi cells transiently expressing Glo-22F with certain combinations of GPCR and Gαi subunits. Cells were stimulated with 10 µM forskolin along with the indicated concentrations of ligands. For each experiment, forskolin-induced cAMP accumulation was set as 100%. In all figures, symbols and error bars represent the mean and SEM, respectively, of three independent experiments. Each experiment was performed in triplicate. Note that for some data points, error bars are smaller than the size of symbols. **b** Schematic diagram showing the process for calculating RAi values. **c** Heatmap of the LogRAi values for the 11 GPCRs. Colors range from white (LogRAi = −1.5) to red (LogRAi = 0). Receptors are arranged according to the dendrogram of the hierarchical clustering, which was calculated from the coupling profiles.

Because the GloSensor cAMP assay dominantly detects the activation of Gαs over Gαi, we selected 11 GPCRs that are coupled to Gαi but not to Gαs, which include dopamine D2 receptor (D2R), dopamine D4 receptor (D4R), mu-type opioid receptor (MOR), somatostatin receptor type 2 (SSTR2), cannabinoid receptor 1 (CB1), lysophosphatidic acid receptor 1 (LPA1), sphingosine 1-phosphate receptor 1 (S1P1), sphingosine 1-phosphate receptor 3 (S1P3), probable G-protein coupled receptor 34 (GPR34), C–C chemokine receptor type 2 (CCR2), and C-C chemokine receptor type 5 (CCR5). Endogenous ligands including dopamine, serotonin, methionine-enkephalin, somatostatin, lysophosphatidic acid, sphingosine 1-phosphate, lysophosphatidylserine, CCL2, and CCL4 were used for all respective GPCRs except for CB1, for which a high-affinity synthetic ligand (CP-55490) was used because endogenous ligands such as anandamide exhibited poor responses in the assay. For each GPCR, we plotted the cAMP responses from the five Gαi conditions and one Gαi-nontransfected condition (Fig. 4a, Supplementary Fig. 6). The inhibition of cAMP accumulation over titrated ligand concentrations was graphed and fitted with a sigmoidal concentration-response curve, from which the half maximal concentration (*EC*_50_) and maximal response (*E*_max_) values were obtained (Supplementary Table 1). We found divergent patterns of Gαi-subunit coupling among the 11 GPCRs. In D2R, for example, all tested Gαi subunits resulted in equivalent saturating concentrations (corresponding to *E*_max_ values), but the concentration–response curve in the Gαo-expressing condition shifted leftward, which reflects the difference in *EC*_50_ values. The observed Gαo preference of D2R is consistent with previous studies using Sf9 cells and intact Gαi subunits which showed that D2R most effectively couples to Gαo^26,27^. In LPA1, on the other hand, the cAMP-suppressive responses in saturating concentrations were comparable between Gαi1–3 and Gαo, while the response was smaller in Gαz.

To compare Gαi-subunit-coupling selectivity across receptors in a uniform way, we calculated a parameter known as relative intrinsic activity (RAi)^28^. RAi value is a single parameter that considers both *EC*_50_ and *E*_max_. While RAi is commonly used to evaluate ligand bias, we used it to assess receptor’s preference for Gαi subunits. For each sigmoidal curve, we divided *E*_max_ by *EC*_50_ and normalized the resulting *E*_max_/*EC*_50_ values by the maximum *E*_max_/*EC*_50_ value among the five tested Gαi subunits to generate dimensionless relative *E*_max_/*EC*_50_ values (defined as RAi, Fig. 4b). We further log-transformed the RAi value (LogRAi) and used it as a Gαi-subunit-coupling index. We applied hierarchical cluster analysis to classify the GPCRs and showed their Gαi-subunit-coupling index as a heatmap with a clustering tree (Fig. 4c). We observed three groups in the clustering analysis. Cluster 1 (S1P1, CCR2, S1P3, D4R, and MOR) coupled efficiently to Gαz, followed by Gαi1–3 and Gαo. Cluster 2 (D2R) coupled to Gαo, followed by Gαi1–3, then Gαz. Cluster 3 (CB1, CCR5, GPR34, SSTR2, and LPA1) did not show selectivity for specific Gαi subunits. We compared the Gαi-coupling-based clustering with GPCR phylogenetic trees. Based on the amino-acid similarity and ligand grouping of GPCRdb (https://gpcrdb.org), the tested receptors were classified into lipid receptors (LPA1, S1P1, S1P3, CB1), orphan receptors (GPR34), aminergic receptors (D2R, D4R), chemokine receptors (CCR2 and CCR5), and peptide receptors (MOR, SSTR2). Interestingly, this classification is distinct from coupling-based clustering. These results suggest that, from an evolutionary perspective, each GPCR has individually acquired varying Gαi subunit selectivity^29^.

## Discussion

In this study, we established a Gαi-deficient HEK293A cell line, ∆Gαi cells, in order to more effectively study the Gαi subunit-coupling preference of GPCRs. Researchers have previously used PTX to inhibit endogenously expressed Gαi family proteins. However, PTX-insensitive mutants are required to assess individual Gαi subunit signaling in PTX-treated conditions. Furthermore, PTX is incapable of inhibiting Gαz. An *Escherichia coli* pertussis toxin-like AB_5_ toxin called OZITX was recently found to inhibit all Gαi subfamily proteins, including Gαz^30,31^. Even with this toxin, a Gαi C-terminal mutation, which may affect coupling properties, is required to make Gαi subfamily proteins insensitive to OZITX. The ∆Gαi cells overcome these experimental problems and allow for investigation of not only GPCR-Gαi subunit coupling but also downstream signaling of intact, individual Gαi subunits.

The coupling preference of D2R for Gαo as well as D4R for Gαz provides signaling properties suitable for physiological functions. Gαz has a slow spontaneous GDP dissociation rate as well as low intrinsic GTPase activity^11^. D4R is expressed in the retina and regulates the circadian nature of light-adapted vision^32,33^. Both D4R and Gαz are expressed in photoreceptor cells and display a daily expression pattern that peaks at night^34^. Efficient coupling of D4R and Gαz may allow for regulation of the circadian nature of vision by taking advantage of the long-term activation properties of Gαz. Although D2R is phylogenetically the most similar to D4R and together comprise D2-like receptors, D2R is efficiently coupled to Gαo. In contrast to Gαz, Gαo has a faster spontaneous GDP dissociation rate than the other Gαi subunits^10^. Therefore, it is likely that D2R possesses fast-responding signaling properties through Gαo, which may allow for rapid signal transduction between neurons. Altogether, D2R and D4R likely utilize the differential coupling selectivity to allow for appropriate duration of dopaminergic signaling outcomes. Since signaling properties of GPCRs are also dependent on Gα expression profiles of the cells and their localization, which are different among cell types, signaling properties of D2R and D4R in vivo need to be further investigated in the future studies.

MOR is an important drug target for analgesics. In a previous study using PTX and PTX-insensitive Gαi subunits, MOR was coupled more effectively to Gαi3 and Gαo than to Gαi1 and Gαi2^14^. However, in our experimental conditions, MOR couples almost comparably to Gαi1-3 and Gαo. Although difference in the amount of Gα proteins between the two studies could also affect coupling, this observed difference may be due to the use of the C-terminal mutation in the Gαi subunits in the previous study in order to confer resistance to PTX, suggesting the importance of evaluating Gαi subunit selectivity using intact Gαi subunits. Additionally, we measured the coupling of MOR for Gαz in parallel with Gαi1-3 and Gαo without modifications in the α subunits. In vivo experiments have revealed differential roles of Gαi family members in morphine-induced analgesic effects: Gαz in chronic administration^35,36^ and Gαi2 and Gαo in acute analgesic action^37,38^. In consideration of biased ligands, which induce distinct effector activation on the same receptor^39–41^, a Gαz-biased MOR agonist could potentially lead to the development of a long-acting analgesia with possibly lower tolerance. Altogether, evaluating the Gαi subunit selectivity of MOR agonizts may ultimately enhance our understanding of the differences in drug activity and contribute to the discovery of more desirable analgesics.

Along with our previously generated ∆Gαs, ∆Gαq, and ∆Gα12 cells^42–44^, we now have a full set of the four Gα knock-out cell lines. These cell lines can be used to investigate Gα signaling downstream of GPCRs individually, which will increase our understanding of GPCR signaling^45^ and ultimately allow for the development of drugs with more optimal therapeutic effects.

## Methods

### Reagents and plasmids

MetEnk (4042-v) and somatostatin (4023-v) were purchased from Peptide Institute. S1P (62570) and CP-55490 (90084) were purchased from Cayman Chemical. Dopamine (040-15433) was purchased from FUJIFILM Wako Pure Chemical. LPA (867130) and LysoPS (858143) were purchased from Avanti Polar Lipid. CCL2 (Z03292) and CCL4 (Z02831) were purchased from Genscript. Plasmids used in Glosensor cAMP assay^3^ and TGFα shedding assay^25^ were previously described.

### Cell culture and transfection

HEK293A cells (Thermo Fisher Scientific) and their derivative ΔGαi cells were maintained in Dulbecco’s Modified Eagle Medium (DMEM, Nissui Pharmaceutical) supplemented with 10% fetal bovine serum (Gibco, Thermo Fisher Scientific), 0.006% (w/v) penicillin and 0.01% (w/v) streptomycin (complete DMEM), and maintained at 37 °C in humidified incubator equilibrated with 5% CO_2_. Transfections were performed by using polyethylenimine (PEI) solution (Polyethyleneimine “Max”, Polysciences). Typically, HEK293 cells were seeded in a 6-well culture plate at cell density of 2 × 10^5^ cells per mL in 2 mL of the complete DMEM and cultured for one day in a CO_2_ incubator. A transfection solution was mixed by combining plasmid solution diluted in 100 µl of Opti-MEM (Life technologies) and 4 µl of 1 mg per mL PEI solution in 100 µl of Opti-MEM. The transfected cells were further incubated for one day before subjected to an assay as described below.

### Generation of ∆Gαi-HEK293 cells

The ΔGαi cells were obtained by the iterative CRISPR/Cas9-mediated mutagenesis strategy illustrated in Fig. 1B. sgRNA constructs targeting the *GNAI1*, *GNAI2*, *GNAI3*, *GNAO*, *GNAZ*, *GNAT1*, and *GNAT2* genes were designed using the CRISPR.MIT.EDU website (already shut down) so that a SpCas9-mediated DNA cleavage site (3-bp upstream of the protospacer adjacent motif (PAM) sequence) encompasses a restriction enzyme-recognizing site. Sense and antisense oligonucleotide encoding the guide RNA was synthesized (FASMAC) and inserted into the BbsI site of the pSpCas9 (BB)-2A-GFP (PX458) vector (a gift from Feng Zhang, Broad Institute, Cambridge, MA; Addgene plasmid No.48138). Correct insertion of the guide RNA sequences was verified by sequencing using the Sanger method (FASMAC). HEK293A cells were seeded into 6-well plates (1.0 × 10^5^ cells per mL in 2 mL of complete DMEM) and 24 hours later, transfected with a combination of PX458 vectors targeting the Gαi subunit encoding genes using Lipofectamine 2000 (Thermo Fisher Scientific). Three days later, the cells were harvested and top 10–20% of GFP-positive cells were isolated by fluorescence-activated cell sorter (SH800; SONY). The isolated cells were distributed in 96-well plates for clonal expansion. Clones were analyzed for mutations in target genes by PCR and restriction enzyme digestion using capillary electrophoresis system (MultiNA, Shimadzu). The restriction enzyme-resistant PCR products were assessed for their genomic DNA alterations by direct sequencing or TA cloning. Successful targeting was confirmed by assessing GPCR-mediated cAMP inhibition, as described below. Oligonucleotide sequences encoding guide RNAs, PCR primers used to amplify the sgRNA-targeting sites, and the restriction enzymes used to digest PCR products were listed in Supplementary Table 2.

### Quantitative real-time PCR analysis

Total RNA from HEK293 cells was prepared using a GenElute Mammalian Total RNA Miniprep kit (Sigma-Aldrich). Total RNA was reverse-transcribed using High-Capacity cDNA RT kits (Applied Biosystems) according to the manufacturer’s instruction. Real-time quantitative PCRs were performed with TB Green Premix Ex Taq II (Tli RNaseH Plus) (Takara Bio) and monitored by ABI Prism 7300 (Applied Biosystems). RNA expression data were normalized to the expression of *GAPDH*. The PCR primer sequences were listed in Supplementary Table 3.

### GloSensor cAMP assay

Plasmid transfection was performed in a 6-well plate with a mixture of 1 µg (per well in a 6-well plate) Glo-22F cAMP biosensor-encoding pCAGGS plasmid (gene synthesized with codon optimization by Genscript), 200 ng of GPCR-encoding plasmid and 100 ng of Gαi subunit-encoding plasmid. After 1-day incubation, the transfected cells were harvested with 0.53 mM EDTA-containing D-PBS, centrifuged at 190 g for 5 min and suspended in 0.01% bovine serum albumin (BSA, fatty acid-free and protease-free grade, Serva) and 5 mM HEPES (pH 7.4) containing Hank’s Balanced Salt Solution (HBSS). The cells were seeded in a white 96-well plate (30 µl per well) and loaded with D-luciferin potassium solution (10 µl of 8 mM solution per well; FujiFilm Wako Pure Chemical). After 2 hour incubation in the dark at room temperature, the plate was read for its initial luminescent count (Spectramax L, Molecular Devices). The cells were treated with a ligand alone (Gs assay) or 10 µM forskolin (FujiFilm Wako Pure Chemical) together with a ligand (Gi assay) (10 µl of 5× solution per well). For kinetics measurement, the plates were measured for 20 min and expressed as fold change values. Fold-change luminescent signals from 16 min to 20 min after compound addition were averaged and normalized to those in forskolin-treated condition. Using Prism 9 software (GraphPad Prism), the cAMP signals were fitted to a four-parameter sigmoidal concentration–response curve, from which *EC*_50_ and *E*_max_ values were obtained. Hierarchical clustering of the coupling profile was performed in python using the Ward method.

### TGFα shedding assay

The TGFα shedding assay was performed as described previously with minor modifications^25^. Plasmid transfection was performed in a 6-well plate with a mixture of 500 ng (per well in a 6-well plate) AP-TGFα-encoding plasmid and 200 ng GPCR-encoding plasmid. After 1-day culture, the transfected cells were harvested by trypsinization, pelleted by centrifugation at 190 g for 5 min and washed once with 5 mM HEPES (pH 7.4)-containing HBSS. After centrifugation, the cells were resuspended in 6 mL of the HEPES-containing HBSS. The cell suspension was seeded in a 96-well culture plate (cell plate) at a volume of 90 µl (per well hereafter) and incubated for 30 min in a CO_2_ incubator. The cells were treated with a GPCR ligand (10×, diluted in HBSS containing 5 mM HEPES (pH 7.4) and 0.01% (w/v) BSA. After spinning the cell plates, 80 µl of conditioned media was transferred to an empty 96-well plate (conditioned media (CM) plate). Totally, 80 µl of AP reaction solution (10 mM *p*-nitrophenylphosphate (*p*-NPP), 120 mM Tris-HCl (pH 9.5), 40 mM NaCl and 10 mM MgCl_2_) was dispensed into the cell plates and the CM plates. Absorbance at 405 nm of the plates was measured, using a microplate reader (SpectraMax 340 PC384, Molecular Devices), before and after 1 hr incubation at room temperature. Ligand-induced AP-TGFα release was calculated by subtracting spontaneous AP-TGFα release signal from ligand-induced AP-TGFα release signal.

### Flow cytometry

Flow cytometry analysis was performed as previously described^3^. Plasmid transfection was performed in a 6-well plate with 1 µg (per well in a 6-well plate) of Glo-22F, 100 ng of Gαi1, and varying amount of plasmid encoding MOR harboring the N-terminal hemagglutinin signal sequence, followed by the FLAG epitope tag and a 15-amino-acid flexible linker. The transfected cells were harvested by adding 300 µl of 0.53 mM EDTA-containing D-PBS, followed by 300 µl of 5 mM HEPES (pH 7.4)-containing HBSS. The cell suspension was dispensed in a 96-well V-bottom plate (200 µl per well, two wells per sample). After centrifugation at 700*g* for 1 min, the cells were washed once with D-PBS and pelleted. Cell pellets were suspended in 2% goat serum- and 2 mM EDTA-containing D-PBS (blocking buffer; 100 µl per well) and incubated for 30 min on ice. After centrifugation at 700*g* for 1 min, the cells were stained with anti-FLAG epitope tag monoclonal antibody (Clone 1E6, FujiFilm Wako Pure Chemicals; 10 µg/mL in the blocking buffer; 50 µl per well) for 30 min on ice. After rinse with D-PBS, the cells were labeled with a goat anti-mouse IgG secondary antibody conjugated with Alexa Fluor 488 (Thermo Fisher Scientific; 10 µg/mL dilution in the blocking buffer; 25 µl per well) for 15 min on ice. The cells were washed once with D-PBS, resuspended in 100 µl of 2 mM EDTA-containing-D-PBS and filtered through a 40 µm filter. The fluorescently labeled cells (approximately 20,000 cells per sample) were analyzed by the EC800 flow cytometer (Sony). Fluorescent signal derived from Alexa Fluor 488 was recorded in an FL1 channel and flow cytometry data were analyzed by a FlowJo software (FlowJo). We used all of the recorded fluorescent signals and calculated mean fluorescence intensities (MFI).

### NanoBiT-miniG recruitment assay

Plasmid transfection was performed in a 6-well plate with a mixture of 500 ng NES-LgBiT-miniGi1-encoding plasmid, whose linker length between LgBiT and miniGi1 was extended to 15-amino acids from the original construct^46,47^, and varying amount of C-terminally SmBiT-fused MOR plasmid (with the N-terminal hemagglutinin signal sequence, followed the FLAG epitope tag and a 15-amino-acid flexible linker). After 1-day culture, the transfected cells were harvested with 1 mL of 0.53 mM EDTA-containing D-PBS, followed by addition of 2 mL the HEPES-containing HBSS. The cells were centrifuged at 190 g for 5 min and resuspended in 2 mL of the 0.01% BSA- and 5 mM HEPES (pH 7.4)-containing HBSS (assay buffer). The cell suspension was seeded in a 96-well culture white plate (Greiner Bio-One) at a volume of 80 µl (per well hereafter) and loaded with 20 µl of 50 µM coelenterazine (Carbosynth) solution diluted in the assay buffer. After 2-hour incubation with coelenterazine at room temperature, MetEnk (6 µM, diluted in the assay buffer) was manually added to the cells (20 µl). Luminescent signals were measured every 40 seconds after ligand addition and average luminescent values of 10–15 min were plotted.

### Statistics and reproducibility

For GloSensor cAMP assay and TGFα shedding assay, three independent experiments were performed in duplicate and triplicate, respectively. For quantitative RT-PCR analysis, three independent experiments were performed in duplicate. Symbols and error bars represent mean values and SEM of indicated numbers of independent experiments, respectively. Concentration–response curves were fitted to all data by the Nonlinear Regression: Variable slope (four parameter) in the Prism 9 with a constraint of the Hill Slope of absolute value less than two. For multiple comparison analysis, we tested statistical significance by two-way ANOVA, followed by the indicated post hoc test using Prism 9.

### Reporting summary

Further information on research design is available in the Nature Portfolio Reporting Summary linked to this article.

## Supplementary information

Supplementary Information

Description of Additional Supplementary Files

Supplementary Data

Reporting Summary

## Data Availability

All data generated or analyzed in study are provided in the Supplementary Data 1.
