# Supplementary material for: Generation of Gαi knock-out HEK293 cells illuminates Gαi-coupling diversity of GPCRs
Source: Commun Biol. 2023 Jan 28;6:112. doi: 10.1038/s42003-023-04465-2 (PMC9884212; doi:10.1038/s42003-023-04465-2)
Supplement: Supplementary file 1 — Supplementary Information [file 42003_2023_4465_MOESM1_ESM.pdf]

Supplementary Figure 1

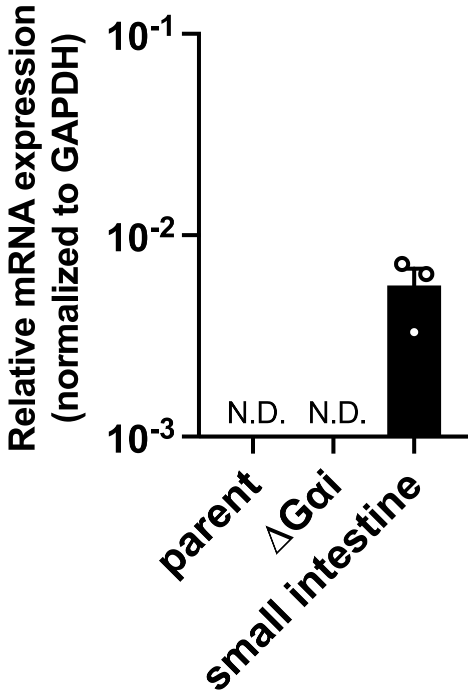

Supplementary Figure 1. *GNAT3* expression in small intestine tissue and ΔGαi cells.

Quantitative real-time PCR analysis of the *GNAT3* gene in parent and the ΔGαi cells as well as small intestine tissue. Bars and error bars represent the mean and SEM, respectively, of three independent experiments. Each experiment was performed in triplicate. Abbreviation: N.D., not detected.

Supplementary Figure 2

GNAI1

Parent 5’ -GGTTGAAGATAGACTTTGGTGACTCAGCCCGGGCGgtaagttattaaatttgttggagct-3’

a. a. 90 R L K I D F G D S A R A 109

ΔGi/o(#1) 5’ -GGTTGAAGATAGACTTTGGTGACTCAGCCCGGGCGgtaagttattaaatttgttggagct-3’
↑C 1-bp insertion

ΔGi/o(#2) 5’ -GGTTGAAGATAGACTTTGGTGACTCAGCCCGGGCGgtaagttattaaatttgttggagct-3’
↑95-bp insertion
AGCTGGTTGTAGGTCTGCACCAGCTGGATGAACAGCTTGTCCACGTCGCTGTTGTCGGGGTTCAG
GTCGCCCTCGATCAGGAAGTGGCCCCTGTC

GNAI2

Parent 5’ -TGCTACGGACCCGCGTAAAGACCACGGGGATCGTGGAGACACACTTCACCTTCAAGGACC-3’

a. a. 175 V L R T R V K T T G I V E T H F T F K D 194

ΔGi/o(#1) 5’ -TGCTACGGACCCGCGTAAAGACCACGGGG-TCGTGGAGACACACTTCACCTTCAAGGACC-3’
1-bp deletion

ΔGi/o(#2) 5’ -TGCTACGGACCCGCGTAAAGACCACGGGGATCGTGGAGACACACTTCACCTTCAAGGACC-3’
↑T 1-bp insertion

ΔGi/o(#3) 5’ -TGCTACGGACCCGCGTAAAGACCACGGGGATCGTGGAGACACACTTCACCTTCAAGGACC-3’
↑283-bp insertion
TGGGTGACGCCTGCACCGACCATCGCCACCAGCGCCAGCCCCTGACGCAGGCGCAGTTCGCCAGG
TAATCCCGCGGCATAGGTTTTCAGCCGTTCCTCGATCATCTCTCTGTCCTCAAACAGTGTCAGGG
STOP
TCAGCACGATATCTTCCAGAATGTCCTCGTTTTCCTCATTGTCCAGGAAGTCCTTGTCCTTGATA
ATTTTCAGCAGATCGTGGTATGTGCCCAGGGAGGCGTTGAACCGATCTTCCACGCCGGAGATTTC
CACGGAGTCGAAGCACTCGATTT

GNAI3

Parent 5’ -ATGGTGGGGTACAAGCTTGCTTCAGCAGATCCAGGGAATATCAGCTCAATGATTCTGCT-3’

a. a. 133 D G G V Q A C F S R S R E Y Q L N D S A 152

ΔGi/o(#1) 5’ -ATGGTGGGGTACAAGCTTGCTTCAGCAGATCCAGGGAATATCAGCTCAATGATTCTGCT-3’
↑T 1-bp insertion STOP

GNAZ

Parent 5’ -ACAAGCCCCTCATCATCTACAATGCCATCGACTCGCTGACCCGCATCATCCGGGCCCTGG-3’

a. a. 69 Y K P L I I Y N A I D S L T R I I R A L 88

ΔGi/o(#1) 5’ -ggcgccgcgtctctttcagGTGCCGGTGAGTCCGGGAAGAGCACCATCGTCAAGCAGATG-3’
↑G 1-bp insertion

Supplementary Figure 2 (continued)

GNAT1

Parent 5’ -ggcgccgcgtctctttcagGTGCCGGTGAGTCCGGGAAGAGCACCATCGTCAAGCAGATG-3’

a. a. 35 G A G E S G K S T I V K Q M 49

ΔGi/o(#1) 5’ -ggcgccgcgtctctttcagGTGCCGGTGAGTCCGGGAAGAGCACCATCGTCAAGCAGATG-3’
↑G 1-bp insertion

GNAT2

Parent 5’ -CCATTGAGGAGGGAACCATGCCTCCTGAGCTCGTGGAGGTCATTAGGAGGTTGTGGAAG-3’

a. a. 113 S I E E G T M P P E L V E V I R R L W K 132

ΔGi/o(#1) 5’ -GGTTGAAGATAGACTTTGGTGACTCAGCCCGGGCGgtaagttattaaatttgttggagct-3’
↑C 1-bp insertion

ΔGi/o(#2) 5’ -GGTTGAAGATAGACTTTGGTGACTCAGCCCGGGCGgtaagttattaaatttgttggagct-3’
↑95-bp insertion
AGCTGGTTGTAGGTCTGCACCAGCTGGATGAACAGCTTGTCCACGTCGCTGTTGTCGGGGTTCAG
GTCGCCCTCGATCAGGAAGTGGCCCCTGTC

GNAO1

Parent 5’ -CTCTGAGCGCAGAGGAGAGAGCCGCCCTCGAGCGGAGCAAGGCGATTGAGAAAAACCTCA-3’

a. a. 4 T L S A E E R A A L E R S K A I E K N L 23

ΔGi/o(#1) 5’ -CTCTGAGCGCAGAGGAGAGAGCCGCCCTCGAGCGGAGCAAGGCGATTGAGAAAAACCTC-3’
↑A 1-bp insertion

ΔGi/o(#2) 5’ -CTCTGAGCGCAGAGGAGAGAGCCGCCCTCGAGCGGAGCAAGGCGATTGAGAAAAACCTC-3’
↑141-bp insertion
ATACCACCTGAGAAAGAAACTGGTGGACAGCACCGACAAGGCCGACCTGCGGCTGATCTATCTG
STOP
GCCCTGGCCCACATGATCAAGTTCCGGGGCCACTTCCTGATCGAGGGCGACCTGAACCCCGACA
ACAGCGACGTGGG

ΔGi/o(#2) 5’ -CTCTGAGCGCAGAGGAGAGAGCCGCCCTCGAGCGGAGCAAGGCGATTGAGAAAAACCTC-3’
↑370-bp insertion
TGCAGAATTGGCGCACGCGCTAAAAACGGACTAGCCTTATTTTAACTTGCTATTTCTAGCTCTA
STOP
AAACAAAAAAGCACCGACTCGGTGCCACTTTTTCAAGTTGATAACGGACTAGCCTTATTTTAAC
TTGCTATTTCTAGCTTTTTCTTTCAGGCGGAAGCCAATGTCGTAATCTTCAGTAAGACTCTGCA
CGTCGAAAGCAATACCGTCACCGTCAGCTAACAGTGCGGTCACGGCGCGGCGGCTGAAACAGGT
GCCGACGCCTGCGCTGGGCACTTGTCCGGCGAGGGCTTCACGCACCGGAACATCTTTGCCATGC
AGCTCTGAAAACTCATCAATGTAAGTCATGCTGGTGAAGTGCGTCCGTTC

Supplementary Figure 2. Genomic sequences of the ΔGαi cells.

The sgRNA-target sequences are underlined, and the SpCas9 PAM sequences (NGG) are highlighted in blue. Capital and lowercase letters represent exons and introns, respectively. Red arrowheads indicate putative double-stranded break sites. Restriction-enzyme sites (Sma I (*GNAI1*), Mbo I (*GNAI2* and *GNAI3*), Hinf I (*GNAT1*), Sac I (*GNAT2*), and Tac I (*GNAO*)) are boxed.

Supplementary Figure 3

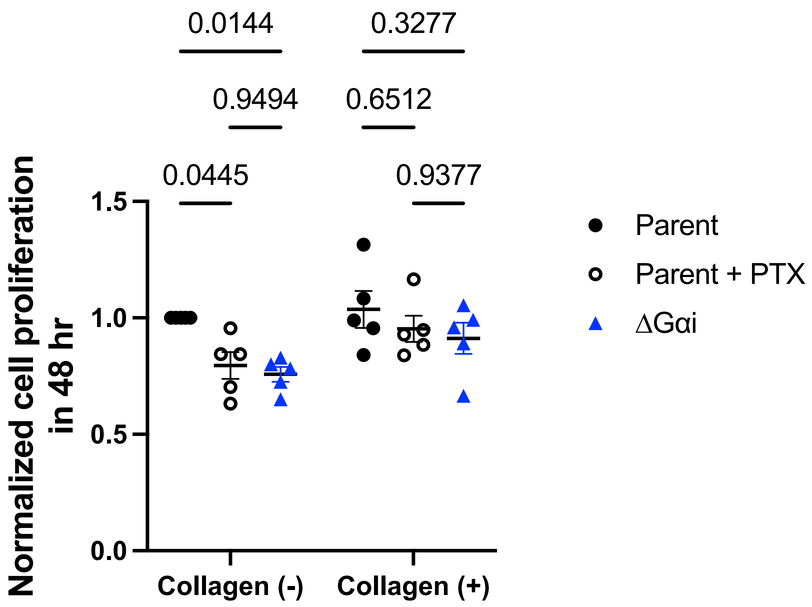

Supplementary Figure 3. Cell proliferation of the parent and the ΔGαi cells.

Cell numbers were counted 48 hours after seeding on a regular cell culture dish or collagen type I-coated dish. For PTX treatment, 100 ng/ml of PTX was added to culture media an hour after cell seeding. Symbols and error bars represent the mean and SEM, respectively, of five independent experiments with each normalized to cell numbers in the parent cell condition. Statistical significance was determined by two-way ANOVA followed by Sidak's post hoc test. *$P < 0.05$ compared with the corresponding control group.

Supplementary Figure 4

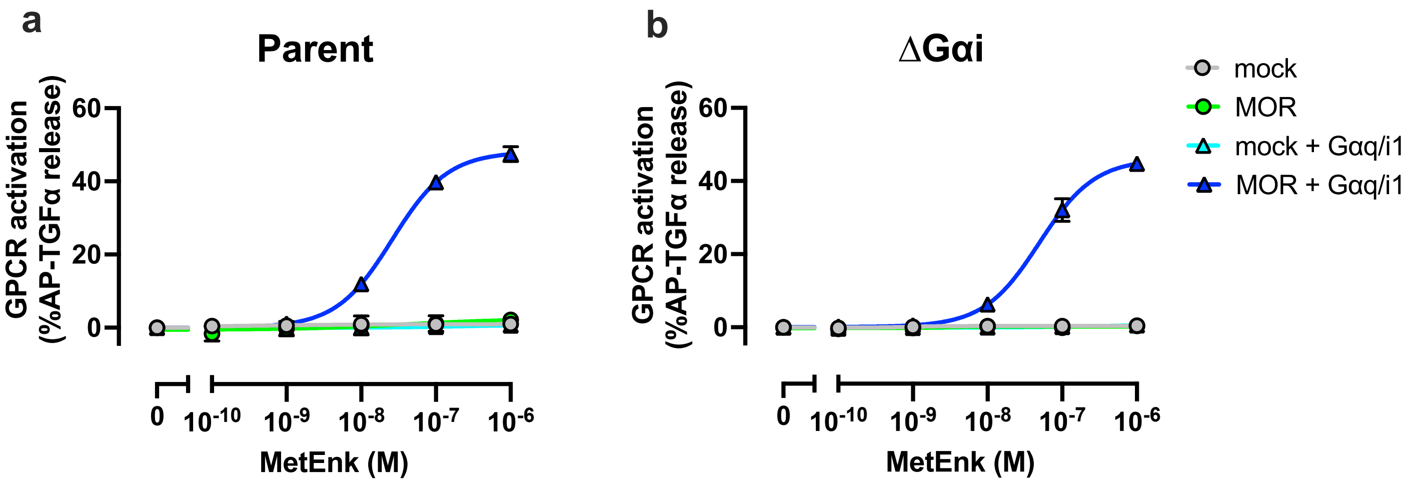

Supplementary Figure 4. Lack of Gq and G12 signaling in the MOR-expressing parent and the ΔGαi cells.

a, b, Concentration-response curve of the TGFα shedding responses induced by MOR activation in parent (a) and the ΔGαi cells (b). MOR was expressed in both cell lines along with the AP-TGFα reporter, and the resulting ligand-induced response was assessed. As a positive control, we used co-expression of the chimeric Gαq/i1 subunit. In all figures, symbols and error bars represent the mean and SEM, respectively, of three independent experiments. Note that for many data points, error bars are smaller than the size of the symbols, and thus are not visible.

Supplementary Figure 5

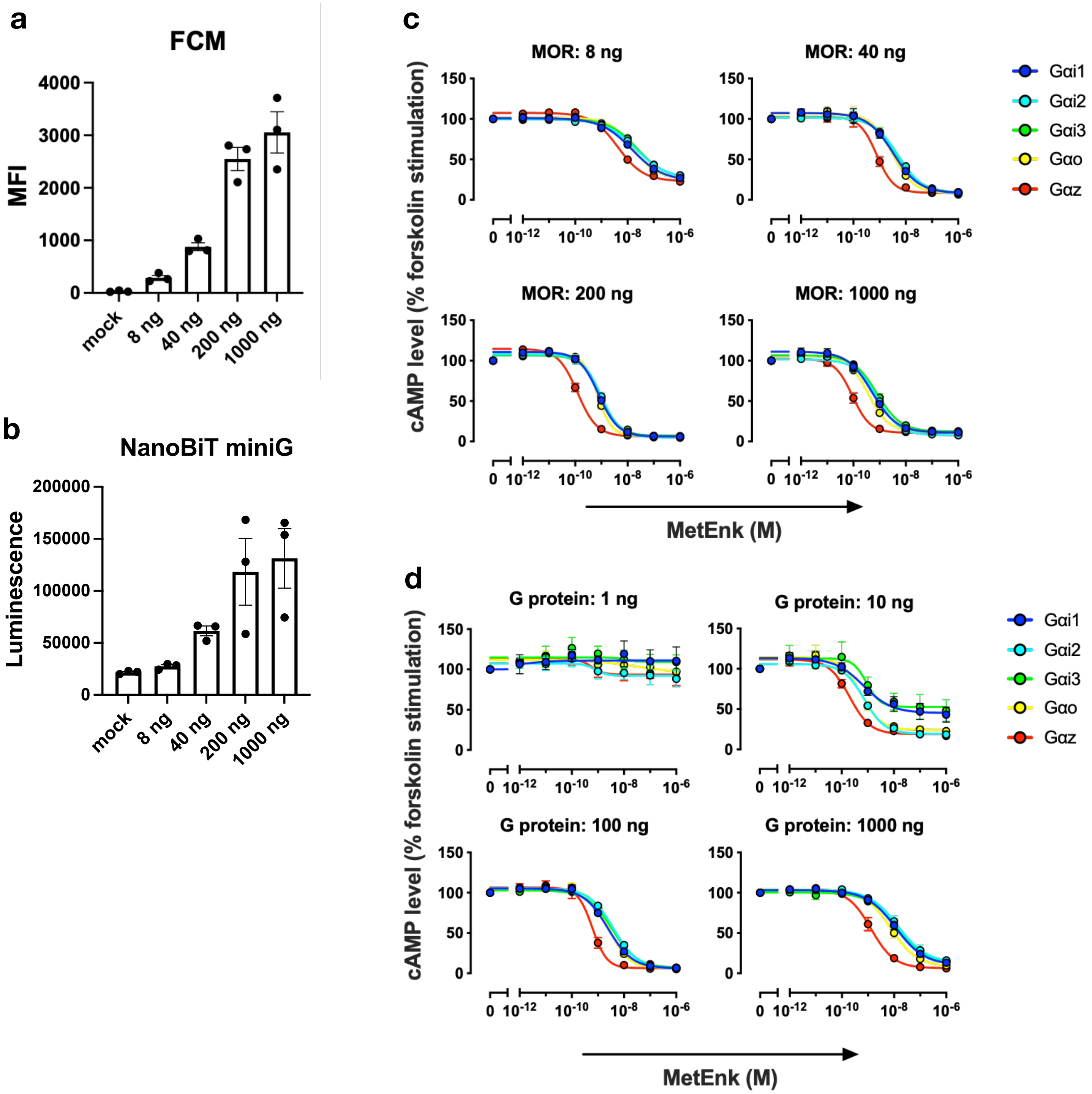

Supplementary Figure 5. Effect of transfection amounts of plasmids encoding receptor and Gα on cAMP responses.

a, Flow cytometry analysis of MOR expression. Indicated amounts (per well in a 6-well plate) of N-terminally FLAG epitope-tagged MOR were transfected together with 100 ng of Gαi1 and 1 µg of Glo-22F into the ΔGαi cells. The transfected cells were labeled with anti-FLAG tag antibody, followed by a secondary antibody conjugated with Alexa Fluor 488, and subjected to flow cytometry analysis. Mean fluorescence intensity (MFI; arbitrary units) of three independent experiments are shown. Symbols and error bars represent the mean and SEM, respectively.

b, NanoBiT miniG recruitment assay of MOR. Indicated amounts (per well in a 6-well plate) of C-terminally SmBiT-fused MOR were transfected together with 500 ng of N-terminally LgBiT-fused miniGi1 into the ΔGαi cells. Luminescent counts (arbitrary units) after 1 µM MetEnk stimulation was shown. Symbols and error bars represent the mean and SEM, respectively, of three independent experiments.

c, d, Concentration-response curves of GloSensor cAMP assays in the ΔGαi cells transiently expressing Glo-22F with varying amounts of MOR (c) and Gαi subunits (d). Cells were stimulated with 10 µM forskolin along with the indicated concentrations of ligands. For each experiment, forskolin-induced cAMP accumulation was set as 100%. In all figures, symbols and error bars represent the mean and SEM, respectively, of three independent experiments. Each experiment was performed in duplicate. Note that for some data points, error bars are smaller than the size of symbols.

Supplementary Figure 6

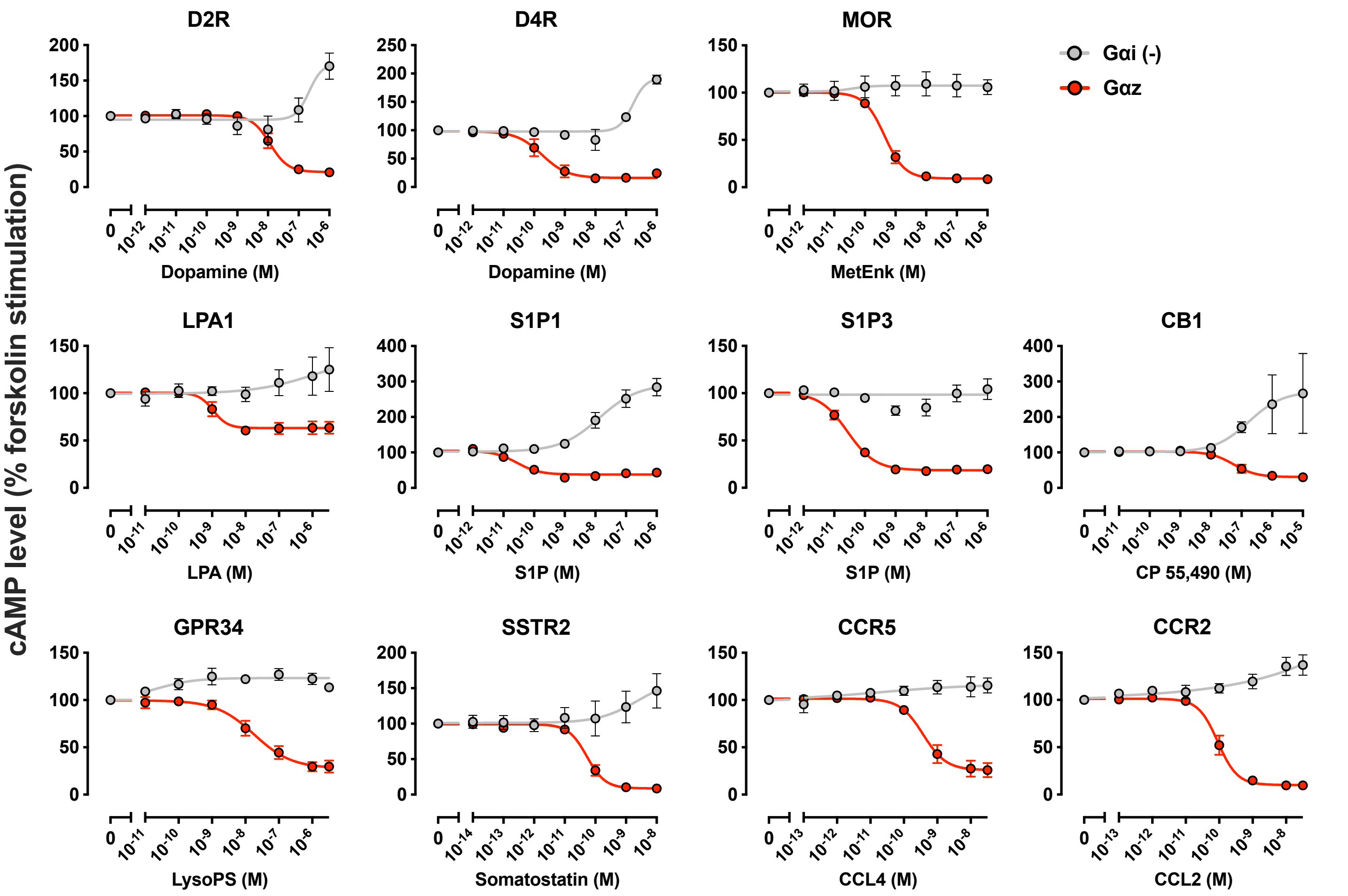

Supplementary Figure 6. Ligand-induced cAMP levels in ΔGαi cells without exogenous Gαi expression.

Concentration-response curves of GloSensor cAMP assays in the ΔGαi cells transiently expressing Glo-22F and GPCR of interest with or without Gαz subunits. Cells were stimulated with 10 μM forskolin along with the indicated concentrations of ligands. For each experiment, forskolin-induced cAMP accumulation was set as 100%. In all figures, symbols and error bars represent the mean and SEM, respectively, of three independent experiments. Each experiment was performed in triplicate. Note that for some data points, error bars are smaller than the size of symbols and the data points of Gαz-expressing condition are the same as in Figure 4a.

Supplementary Table 1

Receptor	n	Gαi1		Gαi2		Gαi3		Gαo		Gαz	
		Emax	pEC50	Emax	pEC50	Emax	pEC50	Emax	pEC50	Emax	pEC50
D2R	3	85.3 ± 2.3	8.48 ± 0.07	90.1 ± 1.9	8.44 ± 0.04	78.9 ± 2.7	8.24 ± 0.09	80.3 ± 1.8	9.09 ± 0.04	80.2 ± 5.6	7.91 ± 0.12
D4R	3	36.2 ± 3.8	8.87 ± 0.10	32.0 ± 3.8	8.81 ± 0.24	32.5 ± 5.3	8.89 ± 0.13	64.3 ± 2.3	8.87 ± 0.10	83.1 ± 2.9	9.74 ± 0.31
MOR	3	87.3 ± 3.1	8.32 ± 0.06	90.0 ± 2.3	8.26 ± 0.06	87.4 ± 1.8	8.13 ± 0.09	87.7 ± 2.3	8.50 ± 0.05	91.0 ± 3.9	9.37 ± 0.11
LPA1	3	69.2 ± 4.2	8.43 ± 0.14	72.1 ± 2.4	8.51 ± 0.12	68.3 ± 2.3	8.52 ± 0.14	62.2 ± 3.1	8.32 ± 0.17	38.0 ± 6.9	9.08 ± 0.35
S1P1	3	75.9 ± 2.7	9.67 ± 0.11	80.6 ± 2.1	9.61 ± 0.16	73.3 ± 2.9	9.85 ± 0.10	57.5 ± 5.8	9.85 ± 0.10	64.2 ± 3.2	10.50 ± 0.15
S1P3	3	82.9 ± 1.5	9.44 ± 0.10	86.7 ± 0.7	9.52 ± 0.16	82.7 ± 1.6	9.59 ± 0.12	79.4 ± 0.4	9.34 ± 0.09	82.0 ± 0.6	10.57 ± 0.09
CB1	3	75.1 ± 4.0	7.49 ± 0.25	81.8 ± 3.2	7.63 ± 0.24	69.2 ± 6.7	7.54 ± 0.26	65.0 ± 3.6	7.32 ± 0.26	71.2 ± 6.2	7.28 ± 0.19
GPR34	3	50.4 ± 5.4	7.77 ± 0.16	51.4 ± 5.4	7.70 ± 0.20	50.2 ± 6.2	7.75 ± 0.23	50.2 ± 4.9	7.82 ± 0.21	72.3 ± 3.6	7.77 ± 0.14
SSTR2	3	88.9 ± 4.4	10.37 ± 0.12	77.6 ± 10.7	10.18 ± 0.14	83.9 ± 1.7	10.40 ± 0.06	85.5 ± 2.6	9.94 ± 0.15	90.5 ± 2.6	10.31 ± 0.11
CCR2	3	82.0 ± 1.8	9.43 ± 0.11	79.6 ± 1.9	9.48 ± 0.09	81.3 ± 0.4	9.67 ± 0.10	71.3 ± 2.1	9.29 ± 0.11	91.7 ± 0.8	10.03 ± 0.15
CCR5	3	61.6 ± 7.1	9.52 ± 0.16	64.8 ± 6.9	9.58 ± 0.12	56.0 ± 9.0	9.56 ± 0.19	58.6 ± 6.0	9.32 ± 0.15	76.0 ± 7.9	9.40 ± 0.09

Supplementary Table 1. E_{max} and EC_{50} values obtained from the sigmoidal curves shown in Figure 3A.

Ligand-induced cAMP responses were fitted to a sigmoidal curve, from which E_{max} (% inhibition of forskolin stimulation) and EC_{50} values were obtained. Mean and SEM values were calculated from three independent experiments. Each experiment was performed in triplicate.

Supplementary Table 2

Gene	Sense	Anti-sense
GNAI1	CACCGCTTTGGTGACTCAGCCCGGG	AAACCCCGGGCTGAGTCACCAAAGC
GNAI2	CACCGCGTAAAGACCACGGGGATCG	AAACCGATCCCCGTGGTCTTTACGC
GNAI3	CACCGAGCTTGCTTCAGCAGATCCA	AAACTGGATCTGCTGAAGCAAGCTC
GNAT1	CACCGTTTCAGGTGCCGGTGAGTCC	AAACGGACTCACCGGCACCTGAAAC
GNAT2	CACCGAACCATGCCTCCTGAGCTCG	AAACCGAGCTCAGGAGGCATGGTTC
GNAO1	CACCGAATCGCCTTGCTCCGCTCGA	AAACTCGAGCGGAGCAAGGCGATTC
GNAZ	CACCGATGCGGGTCAGCGAGTCGA	AAACTCGACTCGCTGACCCGCATC

Gene	Forward primer	Reverse primer	Product (bp)	RE
GNAI1	AGCTGGTTATTCAGAAGAGGAGTG	TGGTCCTGATAGTTGACAAGCC	231	SmaI
GNAI2	CTACCTGAACGACCTGGAGC	AGGTGGTGAGGATGTGCTTG	271	MboI
GNAI3	AGCTGGCAGTGCTGAAGAAG	TCATACAAATGACCAAGGGCTC	304	MboI
GNAT1	TAGGTGTGGCTACGGGGTC	GCACTCTTCCAGCGAGTACC	316	HinfI
GNAT2	ACTGCTTCCATCTTAGGTCTTCG	CATCAACCCACCCTCTCACC	310	SacI
GNAO1	CGACATTTTTGTTTCCAGCCC	GGTCCTTACCGAGCAGGAG	273	XhoI
GNAZ	CGAAATCAAGCTGCTCCTGC	TGTCCTCCAGGTGGTACTCG	359	HinfI

Supplementary Table 2. Oligonucleotide sequences used to generate the ΔGαi cells.

Supplementary Table 3

Gene	Forward primer	Reverse primer	Product (bp)
GAPDH	GCCAAGGTCATCCATGACAACT	GAGGGGCCATCCACAGTCTT	95
GNAI1	TGAAGATGAAGAAATGAACCGAATG	TCTGGATAGCATATAGTGAGAGGG	162
GNAI2	TTGCTGCTGTTGGGTGCTG	TGGACTGGATGGTGTTGCTG	136
GNAI3	GACCACAGGCATTGTAGAAACAC	ATTCGGTTCATCTCCTCGTCC	189
GNAT1	CGTGACCTGCATCATCTTCATC	GTAGGTGTTGGGTCCATCGTAG	238
GNAT2	AGGAGGTTGTGGAAGGATGG	GGTTCAGGTAGTAAGATGCGGAG	91
GNAT3	ATCCCAGAAGTGCAGAGGAC	GCACATACCCAGATGCTGTTATTC	216
GNAO1	ACAGCAACACTATCCAGTCCC	CGTCAGCCTTTCTCTCCTTATC	90
GNAZ	AGGTCAGAGCGCAAAAAGTG	TCCGACTTGTCTGGTTATCCTC	115

Supplementary Table 3. Oligonucleotide sequences used in the RT-PCR analysis.
